# American Association of Obstetricians and Gynecologists

**Published:** 1893-07

**Authors:** 


### Society Proceedings.

### AMERICAN ASSOCIATION OF OBSTETRICIANS AND GYNECOLOGISTS.

*Sixth Annual Meeting, held at Detroit, Mich., June 1, 2, and 3, 1893.*

(*Abstract.*)

[*From the Medical News.*]

### FIRST DAY—JUNE 1ST.

DR. J. HENRY CARSTENS, of Detroit, delivered an address of welcome, which was responded to by DR. GEO. H. ROHÉ, of Baltimore.

Dr. GEORGE F. HULBERT, of St. Louis, read a paper, entitled Intrauterine Asphyxia, with Report of Three Cases. The first case was delivered by natural means, the other two with instrumental assistance. In all, the fetal heart sounds had been heard distinctly half an hour before delivery, but somewhat weakened in force. In all, at the time of delivery, the appearance presented by the children was one of extreme pallor, with a deepened tinge of the lips and absolute muscular relaxation. The heart beat at the rate of from forty to fifty per minute. The usual methods of artificial respiration were resorted to, to the extent of introducing a catheter into the trachea. That air did penetrate was evidenced by the slight crepitus heard during compression of the chest in the expiratory part of the respiratory act. Artificial respiration was maintained until the heart ceased beating—until it ceased to respond to the stimulus presented by the aëration of the blood by the artificial respiration. In none of the cases were there any external evidences of pathological conditions. The umbilical cords did not exceed the normal limits, but in all three cases blood-clots were found occupying the placental surface in more than half of its area. These clots were well formed, intimately attached to the placental tissue, and smooth upon the uterine side. There was nothing to indicate that the clot had been torn from the uterine surface, but rather that placental separation had taken place and the clot had formed and become adherent to the placental tissue. So far as the mothers were concerned, they were in average good health; the first was a primipara, the last two multiparæ. The labors had not been more than twelve hours in duration, and presented no abnormality other than that in the first case the delivery was of the "piston-rod" variety, the head being forced down to the perineum at each pain, and promptly receding to the brim upon the disappearance of the pain. In none of the cases were the pains sustained and vigorous, but rather short and inefficient. In none was chloroform used, and in only one, the first, a rectal injection of twenty grains of chloral was given during the first stage. The element of compression upon the head was not at all pronounced, and would not have attracted attention. In the last two cases there was no pulsation appreciable in the cord; in the first case, no record of this fact had been made. The children were separated from the mothers immediately, and in the first a small amount of blood was permitted to escape from the severed end of the cord. Unfortunately, in none of the cases was a post-mortem examination obtained. There was only a moderate amount of amniotic fluid following the birth of each child. In the first two cases, dilatation was well advanced before the membranes ruptured; in the last case, rupture occurred at the beginning of dilatation.

Dr. J. Henry Carstens, of Detroit, Mich., related a case similar to those reported, and attributed the condition of the child to either large doses of ergot, given by an irresponsible practitioner, or to some disturbance of the centers of respiration.

Dr. John M. Duff, of Pittsburg, expressed the view that in the case of stillborn children,—when, apparently, before birth they were well, the heart beating properly, etc.,—death is due to cerebral hemorrhage. He has seen two post-mortem examinations within the past two years corroborative of this view.

Dr. William H. Wenning, of Cincinnati, read a paper on Placenta Previa. He pointed out that the pathology of placenta previa is far from being definitely settled, owing to the discrepancy between clinical phenomena and anatomic demonstrations. The term was originally employed to express the fact of the after-birth lying before, which might apply to a prolapse of the placenta as well as a primarily low insertion. At the present day it implies a fixation of the placenta to the lower pole of the uterine cavity. Omitting minor points of discussion, the placenta may be said to be previa when it is inserted wholly or in part to that portion of the lower uterine segment which is subject to distention in pregnancy or labor.

Dr. John Milton Duff read a paper, entitled The Care of Pregnant Women. He said that every pregnant woman needs moral and hygienic, if not medical, treatment, and from the time that pregnancy is recognized until convalescence from the lying-in.

Before attending a woman in labor, the physician should be familiar with her personal and family history (medically speaking), so that he may note personal or family idiosyncrasies ; discover and treat diseased conditions, if present ; determine the presentation, if not the position, of the child *in utero* ; ascertain the dimensions of the pelvis, the presence of deformities, and the general physical condition ; and judge of her ability to withstand the throes of labor—in short, so that he may be able to anticipate any accident or abnormal condition that might arise during labor, and, if possible, to ward it off or treat it promptly, scientifically, and successfully.

Attention was called to strychnine as an adjuvant in the treatment of disorders of pregnancy, and as an aid in parturition.

Good results were reported from the systematic administration of doses of from one-thirtieth to one-sixtieth of a grain, three times a day, for from two to six weeks prior to the expected onset of labor.

Dr. Edmund M. Pond, of Rutland, Vt., read a paper, entitled Dilatation of the Cervix for Dysmenorrhea, in which he advocated the use of a light Palmer dilator, and packing the uterus when the cervix is elastic; to be repeated, if necessary. In case of failure, a heavy dilator is to be tried. If the cervix is long, conical, and cartilaginous, and dilation and packing have failed, free division from the internal to the external os, dilatation with a light instrument, and introduction of a stem pessary, to be worn from ten to fourteen days, are to be recommended.

Dr. George S. Peck, of Youngstown, O., read a paper on Extra-uterine Pregnancy, and reported several interesting cases.

Dr. Eugene Boise, of Grand Rapids, Mich., read a paper on The Nature of Shock. He concluded that shock is not a paresis, either partial or general, of the vasomotor nerves, but a hyper-irritation of the entire sympathetic system. The skin is pale and livid, from contraction of the arterioles, in consequence of stimulation of their vasomotor nerves. The heart's action is rapid from stimulation of its sympathetic nerve-supply. There is scanty secretion of urine from contraction of the renal arteries, as a result of stimulation of their nerve supply. The skin, though pale and livid, is bathed in perspiration from stimulation of the secretory nerves of the glands. The pupils are dilated from stimulation of their sympathetic nerve-supply. The pulse at the wrist, while rapid and small as would be expected in vasomotor stimulation, is soft and compressible, in consequence of the scanty relaxation or dilatation of the heart. The condition of the heart may not have been actually demonstrated, but may justly be inferred by analogy—reasoning from the action of the uterus under similar conditions. Each contraction of the uterus is normally followed by a period of perfect relaxation. Over-irritation or stimulation of the uterine ganglia or sympathetic nerve-supply causes rapid contraction, with every imperfect relaxation. It is fair to infer the same condition in the heart under similar conditions. Thus the supply of blood to the arteries is scanty and the blood pressure is low. That the condition of the heart is one of stimulation rather than of paresis, may be considered demonstrated by the fact that, in cases of sudden death from severe shock, the heart has been found contracted and empty. Admitting the correctness of this pathology, it follows that the treatment should be conducted on the line of sedation to the sympathetic system, as by amyl nitrite, nitro-glycerin, morphine, and the application of moist heat, *first*, to the surface; *second*, through the long tube in the colon; and *third*, by transfusion of a saline solution at a comparatively high temperature.

### SECOND DAY—JUNE 2D.

Dr. Walter P. Marnton, of Detroit, read a paper, entitled A Contribution to the Pathology of Surgical Diseases of the Gall-bladder.

Dr. Jas. F. W. Ross, of Toronto, followed with a paper on A Few Practical Notes on the Establishment of Anastomosis Between the Gall-bladder and Intestine for Obstruction of the Common Duct, with the Relation of a Case of Obstruction of the Common Duct by a Small Growth.

The President, Dr. L. S. McMurtry, delivered the Annual Address. His subject was The Present Position of Pelvic Surgery. After a brief sketch of the history of ovariotomy, it was pointed out that from its beginning the department of pelvic surgery has been promoted and advanced by the labors of a few men. When the new surgery of the peritoneum was introduced, it was seized upon in a reckless way by the multitude, and for a time its very existence was threatened. The splendid results of the few who were trained for the work by a rigid apprenticeship were made the basis of operations by many wholly unprepared to carry out an exacting and difficult practice. Operations were undertaken upon inadequate and erroneous conceptions of pathologic conditions, and even, indeed, without any definite pathologic data whatever, until in many influential quarters the greatest discredit was thrown upon the work of able and conscientious men, who were masters of pelvic pathology and pelvic surgery.

Dr. Rosenwasser, of Cleveland, Ohio, read a paper, entitled What are the Indications for Abdominal Section in Intra-pelvic Hemorrhage? He offered the following conclusions:

Intra-pelvic hemorrhage may be free into the peritoneal cavity, or primarily or secondarily circumscribed by true or false membranes. Though nearly always due to ruptured ectopic pregnancy, the same surgical principles underlying the treatment of other similar hemorrhages are applicable in intra-pelvic hemorrhage. Such treatment must, therefore, vary according to the conditions dependent on the hemorrhage, and, secondarily, on the original cause of the hemorrhage ; hence, to prevent free intra-pelvic hemorrhage, abdominal section is indicated in all cases of presumably recognized unruptured tubal pregnancy, either as prophylactic, or for the purpose of removing pathologic conditions not otherwise curable. In all cases of free intra-pelvic hemorrhage, from whatever cause, early or immediate section isthe only safe means of averting a fatal termination. In cases of circumscribed, intra-pelvic hemorrhage, section is indicated for the removal of increasing blood-clot and *débris*, whether due to recurrent bleeding or continued growth of a fetus ; and whenever the symptoms indicate decomposition of the blood-clot. Lastly, section is also indicated whenever the pressure of the circumscribed blood-mass causes obstruction of the bowel.

Dr. H. W. Longyear, of Detroit, read a paper, entitled A Plea for Better Surgery in the Closure of the Abdominal Incision. From investigation and observation he has concluded that suppuration of the wound made in abdominal incision can occur with either method, but is much less liable to take place with the buried suture, if properly placed and sealed, than with the *en masse* suture, as, in the latter, pyogenic germs may enter the wound *via* the sutures or their sinuses. In fact, suppuration is often the result of the dragging in of germs attending the removal of the *en masse* suture.

Very thin or very fat walls lead to the development of hernia after abdominal incision, by preventing the accurate coaptation of the aponeuroses, which could only take place with the *en masse* suture, and would be prevented by the use of the buried method. The extra-peritoneal treatment of the stump in either case is bad, and will some day be considered unsurgical, but if the fascia is brought closely around the stump and held there by the buried suture until it forms a solid firm ring around it, the viscera will be less liable to burst through it than if sutured but for nine or ten days, and afterward wedged apart, before firm cicatrization has had time to take place. When the aponeuroses form solid union with the buried suture, no bandage will be required, as they are as strong as before incision. Imperfect closure is very much less liable to occur when the exact method of the buried suture is used, than with the "guess" method of the *en masse* stitch. In one it is known that the fascia is approximated, while in the other there is less certainty. Long incisions are always to be deprecated, but if healed by first intention after the use of the buried suture, hernia cannot occur, as by this method the chance of the interposition of other tissues between the apposed edges of the fascia is obviated. The straining of vomiting, resulting from the nausea of prolonged anesthesia, need not be feared when the buried suture is used, as the fascia cannot be separated by any such force when united with a strong suture. Too early rising is only to be feared after removal of suture; and as the buried suture remains indefinitely to exert its restraining force, this cause has to do only with the *en masse* suture.

Drainage will be required with both methods of suture, but with the increasing tendency to use small tubes, the danger is becoming much less. Large gauze drains should be avoided on this account. The buried suture, however, properly protected from the drainage-opening by one *en masse* stitch, will usually insure solid union of the fascia close to the tube, and thus give the best result. Failure to take up the aponeurosis in the suture can only occur with the *en masse* stitch. Too early removal of sutures can only occur with the *en masse* stitch, and is obviated by using the buried suture. The use of soft catgut is permissible only when the safe tendon suture, or even silkworm-gut or silver wire, cannot be obtained.

If the patient becomes too fat, the cicatrix of the aponeurosis will not separate if reinforced with a strong tendon suture. Non-union of muscles is not likely to occur if their sheaths are properly approximated and held in place by the buried suture. If cut transversely, a fine tendon will insure their union. The use of the interrupted suture is easily obviated by simply using the buried animal suture in the closure of all abdominal incisions.

Dr. C. A. L. REED, of Cincinnati, contributed a paper, entitled The Management of the Abdominal Incision.

He said that the occurrence of suppuration and of ventral hernia in the line of the abdominal incision pointed to some defect of technique or management. In preparation it was recommended that, first, an application of oil be made, followed by ether, with some strong alkali ; then cleansing with clear water, followed by the persistent application, for over half an hour preceding the operation, of a strong solution of mercuric chloride. The incision should be made carefully in the median line through the linea alba, and the peritoneum should immediately be laid open smoothly with the knife. In closure it was recommended that an interrupted suture of silkworm-gut be passed from within outward on both sides entirely through the tissues, but so passed that it enters the wall of the peritoneum near the margin, dips deeply into the median tissue, and is brought out near the margin of the integument. The sutures should not be tied too tightly, and with the knot to one side of the incision. The wound should be dressed with aristol, boric acid, and a bandage carefully applied. When the sutures are removed, a firmly-fitting adhesive strip should be applied that will not cause compression of the incision, with a consequent tendency to separate the internal margin.

THIRD DAY—JUNE 3D.

Dr. JOHN C. SEXTON, of Rushville, Ind., contributed a paper upon The Causation and Prevention of Central Laceration of the Perineum.

He pointed out the rarity of the accident, as a result of the many almost perfect methods of prevention. Among the deformities and irregularities of formation that tend to cause the accident, are defective or abnormal development of the perineum or pubes and anomalies of the expellant forces and positions of the vertex. The use of chloroform to a degree suspending the action of the uterine muscles was advised against as dangerous. Resort to episiotomy was especially cautioned against. It was insisted that timely and skilful use of the forceps could prevent central laceration of the perineum.

Dr. X. O. Werder, of Pittsburg, contributed a paper, entitled Pregnancy Following a Partial Supra-pubic Hysterectomy, Complicated by Hemorrhage through the Abdominal Cicatrix.

The interesting points of the case reported were:

The discharge of the menstrual flow through a small fistulous opening remaining after a partial supra-vaginal hysterectomy; and the occurrence of pregnancy eight or nine weeks after the operation in a woman previously sterile.

The pregnancy terminated at about term in the delivery of a healthy, though poorly developed child, in spite of a firm and unyielding fixation of the uterus to the abdominal wall, and in spite of frequent and profuse hemorrhage from the placental site through the sinus opening in the lower angle of the abdominal cicatrix.

Dr. George H. Rohé, of Catonsville, Md., read a paper, entitled Further Observations on the Relation of Pelvic Disease and Psychical Disturbances in Women. He gave the further history of eighteen cases reported last year, in which the uterine appendages of insane women were removed for pelvic disease. Two additional cases have been operated upon. In both, the ovaries were cystic. Of these twenty cases, there was physical recovery from the operation, with improvement of the general health in eighteen; death in two; absolute mental recovery and discharge from the hospital in four; complete physical and partial mental recovery in three; decided mental improvement, but not sufficient to justify discharge from the hospital, in seven. In three, the mental condition remained unchanged, but the physical condition was decidedly better. In one case, the patient was removed from the hospital by friends a few weeks after operation, and placed in another institution, where she is reported to be worse mentally. A case of melancholia with suicidal tendencies was also reported, in which a badly lacerated cervix was repaired, with complete and rapid mental recovery and discharge.

The following officers were elected:

*President*—Dr. George H. Rohé, of Catonsville, Md.; *First Vice-President*—Dr. Walter P. Manton, of Detroit, Mich.; *Second Vice-President*—Dr. George F. Hulbert, of St. Louis, Mo.; *Secretary*—Dr. William Warren Potter, of Buffalo, N. Y.; *Treasurer*—Dr. X. O. Werder, of Pittsburg, Pa.

It was decided to hold the next meeting at Toronto, Canada, at a date to be fixed on by the Executive Council.

